# Glycomacropeptide Protects against Inflammation and Oxidative Stress, and Promotes Wound Healing in an Atopic Dermatitis Model of Human Keratinocytes

**DOI:** 10.3390/foods12101932

**Published:** 2023-05-09

**Authors:** Pamela Gallegos-Alcalá, Mariela Jiménez, Daniel Cervantes-García, Laura Elena Córdova-Dávalos, Irma Gonzalez-Curiel, Eva Salinas

**Affiliations:** 1 Laboratory of Immunology, Department of Microbiology, Center of Basic Science, Universidad Autónoma de Aguascalientes, Av. Universidad # 940, Aguascalientes 20100, Mexico; pamela.g.alcala@gmail.com (P.G.-A.); mayojv@hotmail.com (M.J.); or dcervantesga@conacyt.mx (D.C.-G.); laura.cordova@edu.uaa.mx (L.E.C.-D.); 2 National Council of Science and Technology, Av. de los Insurgentes Sur 1582, Crédito Constructor, Benito Juárez, Ciudad de México 03940, Mexico; 3 Laboratory of Immunotoxicology and Experimental Therapeutics, Unidad Académica de Ciencias Químicas, Universidad Autónoma de Zacatecas, Carr. Zac.-Gdl. Km 6, Zacatecas 98160, Mexico; irmacuriel@uaz.edu.mx

**Keywords:** bioactive peptides, glycomacropeptide, atopic dermatitis, keratinocytes, cytoprotection, immunomodulation, oxidative stress, wound healing

## Abstract

Keratinocytes are actively implicated in the physiopathology of atopic dermatitis (AD), a skin allergy condition widely distributed worldwide. Glycomacropeptide (GMP) is a milk-derived bioactive peptide generated during cheese making processes or gastric digestion. It has antiallergic and skin barrier restoring properties when it is orally administered in experimental AD. This study aimed to evaluate the effect of GMP on the inflammatory, oxidative, proliferative, and migratory responses of HaCaT keratinocytes in an in vitro AD model. GMP protected keratinocytes from death and apoptosis in a dose dependent manner. GMP at 6.3 and 25 mg/mL, respectively, reduced nitric oxide by 50% and 83.2% as well as lipid hydroperoxides by 27.5% and 45.18% in activated HaCaT cells. The gene expression of *TSLP*, *IL33*, *TARC*, *MDC*, and *NGF* was significantly downregulated comparably to control by GMP treatment in activated keratinocytes, while that of *cGRP* was enhanced. Finally, in an AD microenvironment, GMP at 25 mg/mL stimulated HaCaT cell proliferation, while concentrations of 0.01 and 0.1 mg/mL promoted the HaCaT cell migration. Therefore, we demonstrate that GMP has anti-inflammatory and antioxidative properties and stimulates wound closure on an AD model of keratinocytes, which could support its reported bioactivity in vivo.

## 1. Introduction

The skin is the organ responsible for protecting the body from external agents. The epidermis, the skin’s outermost layer, acts as a barrier to prevent or hinder antigen penetration and pathogen invasion. It is structured by keratinocytes at different stages of differentiation, which are responsible for maintaining skin homeostasis [1]. Disruption of epidermal homeostasis due to functional defects, genetic predisposition, and immune dysregulation causes the onset of the atopic dermatitis (AD), one of the most abundant skin diseases worldwide [1,2]. Although its prevalence varies according to the geographical area, AD occurs in up to 34% of children [2]. This disease commonly appears in early childhood and usually resolves before puberty, although it persists in 2–5% of adults [3]. It is identified as the beginning of the atopic march, an epidemiological theory that proposes that early development of AD predisposes patients to other atopic conditions later in life [4]. In addition, AD represents a significant economic burden for the health sector and patient families and is considered an important global health problem [5].

AD is a chronically relapsing skin inflammatory disease that is triggered in susceptible patients after the constant stimulation of the epidermis with environmental antigens. Keratinocytes are actively implicated in the onset, maintenance, and exacerbation of the inflammation in this disease. The dysfunctional immune response in AD is characterized by a Th2-dominance with an increased production of tumoral necrosis factor (TNF)-α, interleukin (IL)-4, -5, -9, and -22 in lesional skin [1]. Thymic stromal lymphopoietin (TSLP), IL-33, thymus- and activation-regulated chemokine (TARC), and macrophage-derived chemokine (MDC) released by keratinocytes participate in the Th2 differentiation, the activation of innate lymphoid cells (ILC)2, and the recruitment of Th2-type lymphocytes to the site of allergic inflammation [6,7,8]. Although Th2 polarization is prevalent in the disease, other cell populations such as Th1 lymphocytes with interferon (IFN)-γ production are increased in the chronic phase [9]. Moreover, oxidative stress has an important role in AD pathogenesis, since has been associated with exacerbated inflammation and keratinocyte apoptosis. Keratinocytes under the Th2 microenvironment present an elevated level of oxidative stress that can lead to lipid peroxidation, protein oxidation, or DNA damage, with dysfunctional consequences to the cells and barrier function impairment [10]. Likewise, some neuropeptides, such as nervous growth factor (NGF) and calcitonin gene-related peptide (cGRP), have been associated with nerve ending elongation, neurogenic inflammation, and the itching sensation (pruritus) characteristic of AD [11]. Environmental antigens stimulate keratinocytes to express NGF and cGRP in a reactive oxygen species (ROS)-dependent manner [10]. Because of the intense pruritus, AD patients scratch lesional areas aggravating skin damage, which is exacerbated because they have an altered process of wound regeneration since type 2 inflammatory cytokines impair keratinocyte ability to proliferate and migrate properly [12]. Transforming growth factor (TGF)-β, which participates in multiple phases of wound healing [13], has lower expression in AD patients [14]. Therefore, keratinocytes are undoubtedly crucial cells in the early stage of type 2 inflammation, inflammation perpetuation, pruritus development, and skin damage in AD.

Unfortunately, there is no cure for AD. Therapies are focused on mitigating the main symptoms of the disease and achieving long-term disease control. The first-line treatments are emollients to repair epidermal barrier and anti-inflammatory therapy with topical corticosteroids or calcineurin inhibitors to control acute exacerbations and maintain remission [15]. The development of new therapies that control disease symptoms and modify underlying inflammatory and oxidative responses in AD has attracted research interest. These disease-modifying treatments might stop the progression of the atopic march if used in early stages of AD.

Recently, the use of naturally occurring bioactive peptides has been widely explored as potential treatments to different pathologies due to their broad safety and effectiveness. In particular, milk is a source of multiple peptides with diverse biological activities [16]. One of these peptides is the glycomacropeptide (GMP), a 64-amino-acid peptide generated in the cheese whey during the cheese-making process or physiologically during milk enzymatic digestion. It is cleaved from the carboxyl-terminal region of bovine κ-casein by chymosin or pepsin, respectively [17]. Numerous in vitro and in vivo studies have attributed important biological functions to GMP [18]. Among them, antioxidant, anti-inflammatory, and anti-allergic activities are of importance to this study. It has been reported that GMP decreases oxidative stress responses in macrophages and hepatocytes in vitro [19,20]. When orally dosed in rat models of AD, it reduces the intensity of the edema, the infiltration of inflammatory cell, the pruritus, and Th2 cytokine expression in AD lesions [21]. Additionally, GMP administration prevents or reverses cutaneous barrier damage by increasing the expression of structural proteins and antimicrobial peptides, and by avoiding epidermal thickening and *Staphylococcus aureus* colonization in affected skin tissue [22]. The action mechanism of orally administered GMP is partially mediated by prebiotic activities on gut microbiota and the production of immunomodulatory molecules, such as short chain fatty acids (SCFAs) [22,23]. Nevertheless, a cutaneous direct effect of GMP cannot be ruled out, since it has been detected in blood after milk or yogurt ingestion [24], and a modulatory activity of oral GMP on skin inflammatory cells, such as mast cells, has been previously documented [23]. GMP could be also formulated in creams or ointments for topical application. Therefore, the evaluation of the biological activities of GMP in an AD model of keratinocyte is of great interest. The aim of this study was to analyze the regulatory capacity of GMP on AD-associated oxidative, inflammatory, and pruritogenic response of human keratinocytes. The effect of GMP on wound closure in an in vitro model of the atopic microenvironment was also studied.

## 2. Materials and Methods

### 2.1. Cell Culture and AD Model of Keratinocyte

HaCaT cells (human skin keratinocytes cell line; CLS Cell Lines Service, 300493) were cultured in Dulbecco’s Modified Eagle Medium (DMEM; Gibco, Grand Island, NY, USA) supplemented with 10% fetal bovine serum (FBS; Gibco, Grand Island, NY, USA), and 1% penicillin/streptomycin (Sigma-Aldrich, St. Louis, MO, USA) at 37 °C in a humidified atmosphere and 5% CO_2_. The cells were harvested from 85–95% confluent monolayer cultures and passaged with the use of 0.25% trypsin (Sigma-Aldrich, St. Louis, MO, USA) and 0.038% EDTA (Promega, Madison, WI, USA). Then, cells were washed and resuspended in 1 mL of complete DMEM. Viability and cell count were assessed by trypan blue exclusion assay. Third to seventh passages of cells were used for experiments.

HaCaT cells were plated for 24 h to obtain confluency and later treated with GMP (0.01–25 mg/mL) before and during stimulation with one of the following substances to develop an AD model of keratinocyte: 2,4-dinitrochlorobenzene (DNCB; Sigma, St. Louis, MO, USA) prepared in 0.1% dimethyl sulfoxide as a trigger for cellular death and oxidative stress; hydrogen peroxide (H_2_O_2_; J.T baker, Phillip Sburg, NJ, USA) as a trigger for cellular death; or IL-4, TNF-α or IFN-γ (all cytokines obtained from PeproTech, Cranbury, NJ, USA) as triggers for inflammatory and pruritogenic gene expression. For this study, LACPRODAN^®^ CGMP-10 (kindly donated by Arla Food Ingredients Group P/S, Viby, Denmark), was used. All the reagents were 0.22 µm filtered before cell culture use.

### 2.2. MTT Assay

The MTT (3-(4,5-dimethylthiazol-2-yl)-2,5-diphenyltetrazole bromide (Sigma, St. Louis, MO, USA) technique determines the tetrazolium salts reduced by the mitochondrial dehydrogenases of living cells [25]. Cytotoxicity and proliferation assays were performed with 3 or 2 × 10^4^ cells incubated with 0.8, 1.6, 3.1, 6.3, 12.5, 25 mg/mL or 0.01, 0.1, 6.3, 25 mg/mL of GMP, respectively, for 12 h in 96-well plates and later stimulated with DNCB 50 µM or H_2_O_2_ 200 µM for 4 h, or with TNF-α/IFN-γ 10 ng/mL mixture for 24 h. When HaCaT cells were incubated with GMP without a later stimulus, concentrations of 0.01, 0.1, 0.8, 1.6, 3.1, 6.3, 12.5, 25 mg/mL were used. Then, the supernatants were removed, and the cell monolayer was incubated with 100 µL of MTT solution (0.5 mg/mL). Four hours later, the formazan crystals were dissolved with 200 µL of isopropanol with 0.04 N HCl. The optical density (OD) of the samples was read at 595 nm and 655 nm (reference) wavelength in a microplate reader (iMarkTM, Bio-Rad, Tokyo, Japan). The cell viability (expressed as percentage) was calculated with the formula: [OD of the test sample/OD control sample] × 100, and the proliferation index as the ratio of the test sample OD compared to the control sample OD.

### 2.3. Cell Apoptosis Assay

Cell apoptosis was evaluated with the ELISA Cell Death Detection ELISAPLUS kit (Roche Diagnostics GmbH, Mannheim, Germany), to detect histone-associated DNA fragments (nucleosomes) in the cytoplasm as an indicator of late apoptosis. The cells (5 × 10^4^) were treated with or without GMP 25 mg/mL for 12 h in 96-well plates and subsequently stimulated with DNCB 50 µM or H_2_O_2_ 200 µM for 4 h. HaCaT cells were lysed using the buffer supplied by the manufacturer, and after centrifugation at 200× *g* for 10 min, supernatants (cytoplasmic fraction) were collected. The ELISA was developed following the supplier’s instructions and the OD was read in a microplate reader at 405 nm wavelength (iMarkTM, Bio-Rad, Tokyo, Japan). Apoptosis levels were represented as nucleosome enrichment factor released into the cytoplasm and calculated as the ratio of OD at 405 nm of the treated cells to that of control cells.

### 2.4. Nitric Oxide Determination

The nitric oxide (NO) levels were determined by the Griess reaction. In this method, the oxidation of NO in an aqueous solution produces nitrite (NO_2_^−^), which in the presence of a diazotizing reagent in acidic media and a coupling reagent forms a stable azo compound of an intense purple color [26]. HaCaT cells (3 × 10^4^) were treated with or without GMP at 6.3 or 25 mg/mL for 12 h in 96-well plates and then stimulated with 50 µM DNCB for 4 h. A volume of 100 µL of supernatant was collected and 50 µL of 1% sulfanilic acid in 5% phosphoric acid was added. Five minutes later, 50 µL of 0.5% α-naphthylamine in 5 N acetic acid was added. After 5 min, samples were read at 490 nm and 655 nm (reference) in a microplate reader (iMarkTM, Bio-Rad, Tokyo, Japan). Organic nitrite levels in cell supernatant were calculated by interpolating into a standard curve generated with NaNO_2_ (0 to 100 µM).

### 2.5. Measurement of Cellular Hydroperoxide Lipids

Hydroperoxide lipids (LOOH) were evaluated using the FOX 2 method with modifications [27]. This method determines the oxidization of ferrous to ferric ions by LOOH in acidic medium, and the later complexation of ferric ions with xylenol orange to produce a stable purple-blue chromophore. Briefly, HaCaT cells (8 × 10^5^) were incubated with or without GMP at 6.3 or 25 mg/mL for 12 h in 6-well plates and then stimulated with DNCB 50 µM for 4 h. After washing, cells were detached, resuspended in 2 mM Tris HCl, sonicated, and frozen. For LOOH quantification, 20 µL of each sample were added to 180 µL of FOX 2 reagent and incubated for 30 min. FOX2 reagent was freshly prepared with solution A (ammonium ferrous sulfate 250 µM in sulfuric acid 25 mM), and solution B (xylenol orange 100 µM and butylated hydroxytoluene 4 mM); both solutions were prepared in 90% *v*/*v* methanol. The OD was measured at 595 nm in a microplate spectrophotometer (iMarkTM, Bio-Rad, Tokyo, Japan). LOOH levels were calculated by interpolating into a standard curve of tert-butyl hydroperoxide (0 to 50 µM) for the content of cells for each well.

### 2.6. RNA Extraction, Reverse Transcription, and qPCR

HaCaT cells (8 × 10^5^) were treated with or without GMP at 6.3 or 25 mg/mL for 12 h in 6-well plates and stimulated with 15 µM DNCB or inflammatory cytokines (TNF-α/IFN-γ 10 ng/mL, IL-4 50 ng/mL) at 12 h or 24 h, respectively. For *TGFB1* expression, GMP was used at 0.01 or 0.1 mg/mL. Total RNA was isolated from 2.4 × 10^6^ cells using TRIreagent (Sigma, St. Louis, MO, USA) and quantified with NanoDrop™ 2000 (Thermo Scientific, Waltham, MA, USA). For cDNA synthesis, reverse transcription was performed from 1 µg of RNA with the RevertAid First Strain cDNA Synthesis kit (Thermo Scientific, Waltham, MA, USA) in a 2720 thermocycler (Applied Biosystems, Foster City, CA, USA) following the manufacturer’s instructions. For real-time quantitative PCR, the Maxima SYBR Green/ROX qPCR Master Mix (2×) kit (Thermo Scientific, Waltham, MA, USA) was used in the StepOne Real-Time PCR system (Applied Biosystems, Foster City, CA, USA). Expression levels were determined with 2^−∆∆Ct^ method [28], using GAPDH as housekeeping gene. Primers sequences are listed in Table 1.

### 2.7. Wound Healing Assay

Cell motility was evaluated with the wound healing assay with HaCaT cells [29]. Cells were cultured on 24-well plates covered with fibronectin (10 µg/mL) and DMEM with 10% FBS to confluency. Subsequently, regular medium was replaced with DMEM with 1% FBS for 12 h to maintain cells under serum starvation conditions. Then, the cells were treated with mitomycin C (5 µg/mL) for 2 h to arrest cell proliferation and subsequently washed with PBS. An artificial wound was carefully generated with a sterile 200 µL pipette tip that scratches the confluent cell monolayer to make a cell-free cross along the vertical and horizontal diameter of the well. Cells were washed to remove cell debris and re-coated with fibronectin in DMEM with 1% FBS. After 1 h, wound margins were photographed (initial time) using the camera C-B10 attached to the inverted microscope IM-3 (Optika, Bg, Italy). Then, the cells were treated with TNF-α/IFN-γ mixture (10 ng/mL) to induce type-2 environment and GMP (0.01, 0.1, 6.3, 25 mg/mL) or epidermal growth factor (EGF, 10 ng/mL, as a positive control), for 72 h. Cell migration into de scraped area was photo-documented at 24 h, 48 h, and 72 h. The micrographs were captured with the 4× objective to analyze the wound area using the public software FIYI and with the 10× objective to take representative images. The percentage of wound closure was determined considering the initial wound area in 4 randomly selected fields per condition. Additionally, cells were detached from wells at 48 h after the scratch, and total RNA extraction was performed to analyze *TGFB1* gene expression by quantitative real time-PCR.

### 2.8. Statistical Analysis

Data were represented as the mean ± SEM. All data were analyzed with GraphPad Prism 8.0 software (Boston, MA, USA). One-way or two-way ANOVA analysis with multi comparison Bonferroni post-hoc test was used to determine statistical significance, stablishing the significance value at *p* < 0.05.

## 3. Results

### 3.1. GMP Does Not Present Cytotoxic Activity on HaCaT Cells

**Figure 1:**
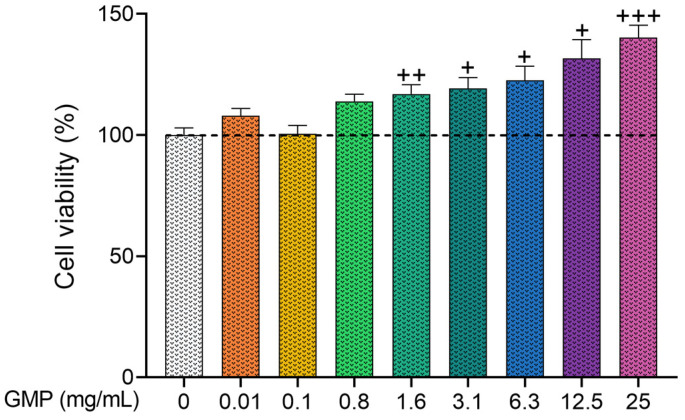
Glycomacropeptide (GMP) has no toxic effect on keratinocytes. HaCaT cells were treated with GMP for 24 h and the percentage of cell viability was determined by 3-(4,5-dimethylthiazol-2-yl)-2,5-diphenyltetrazole bromide (MTT) assay. n = 12, 3 independent experiments in quadruplicate. + *p* < 0.05, ++ *p* < 0.01, +++ *p* < 0.001 vs. control.

First, the viability of human keratinocytes at different concentrations of GMP was analyzed. Results showed that GMP did not have a toxic effect on HaCaT cells at concentrations from 0.01 to 25 mg/mL (Figure 1). GMP at the concentration range between 1.6 and 25 mg/mL appeared to stimulate cell proliferation, as cell viability was 40.3% higher when cells were incubated with 25 mg/mL of GMP compared to the control group (GMP 0 mg/mL). Higher GMP concentrations were not tested due to solubility properties and difficulties in filtering the solution.

### 3.2. Protective Activity of GMP against Cell Death and Apoptosis

**Figure 2:**
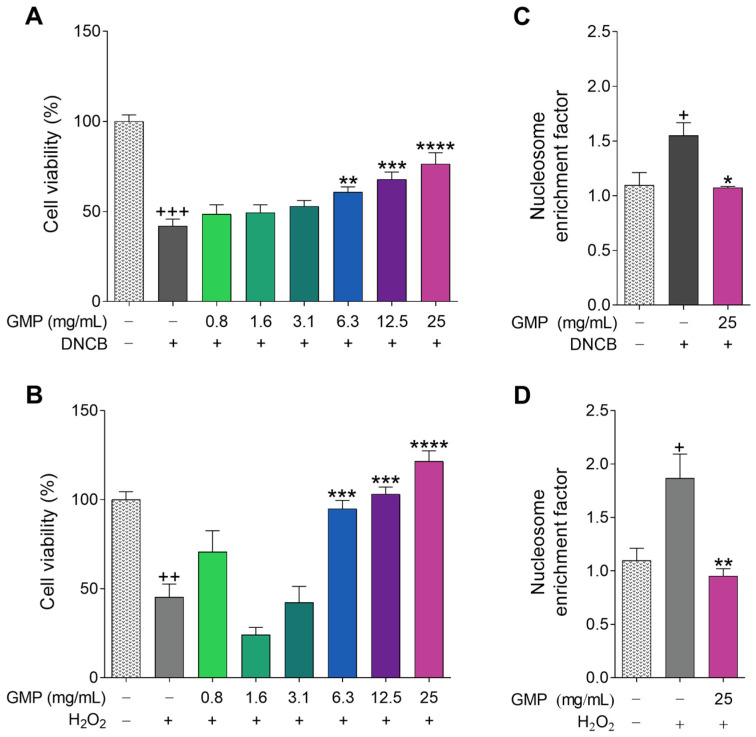
GMP protects keratinocytes against cell death and apoptosis. HaCaT cells were treated with GMP and stimulated with (**A**,**C**) 50 µM 2,4-dinitrochlorobenzene (DNCB) or (**B**,**D**) 200 µM hydrogen peroxide (H_2_O_2_) to determine: (**A**,**B**) the percentage of cell viability by the MTT assay and (**C**,**D**) apoptosis by ELISA. (**A**) n = 9, 3 independent experiments in triplicate; (**B**,**D**) n = 4 independent experiments; (**C**) n = 3 independent experiments. + *p* < 0.05, ++ *p* < 0.001, +++ *p* < 0.0001 vs. control; * *p* < 0.05, ** *p* < 0.01, *** *p* < 0.001, **** *p* < 0.0001 vs. DNCB or H_2_O_2_.

HaCaT cells were incubated with DNCB and H_2_O_2_, two substances that have been reported to induce cell death and apoptosis in keratinocytes [30,31]. Figure 2A shows that DNCB exposure decreased keratinocyte viability by 58%, while GMP treatment exerted a significant cytoprotective effect, increasing cell viability in a dose-dependent manner at concentrations from 6.3 to 25 mg/mL. Likewise, GMP showed the same dose-dependent protective effect when the HaCaT cell death was stimulated with H_2_O_2_ (Figure 2B). This effect could be associated with the proliferative response induced by GMP on cells (Figure 1). We choose the GMP concentration of 25 mg/mL to evaluate its effect on keratinocyte apoptosis. DNCB and H_2_O_2_ increased the level of apoptosis 1.45- and 1.7-fold in HaCaT cells. GMP treatment significantly reduced cell apoptosis to a level similar to that of control conditions, showing a protective role on keratinocyte apoptosis (Figure 2C,D). Based on these results, we decided to use GMP at 6.3 and 25 mg/mL for future experiments.

### 3.3. GMP Protects Keratinocytes from Oxidative Damage

**Figure 3:**
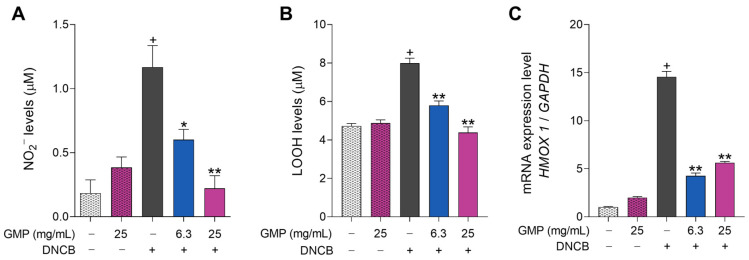
GMP protects keratinocyte against oxidative damage. HaCaT cells were treated with GMP and stimulated with DNCB 50 µM to measure: (**A**) Nitrite (NO_2_^−^) production and (**B**) Lipid hydroperoxide (LOOH) levels; (**C**) HaCaT cells were treated with GMP and stimulated with DNCB 15 µM to analyze HMOX1 gene expression. (**A**,**B**) n = 9, 3 independent experiments in triplicate; (**C**) n = 4 independent experiments. + *p* < 0.0001 vs. control; * *p* < 0.01, ** *p* < 0.0001 vs. DNCB.

As oxidative stress has an important role in AD pathogenesis [10], that eventually causes the death of keratinocytes [31], we evaluated the antioxidant effect of GMP on HaCaT cells. Keratinocytes were stimulated with DNCB to induce oxidative stress and the level of NO secreted by the cells was measured. Cell incubation with DNCB led to a 6.1-fold increase in nitrite production compared to control values, but when cells were GMP-treated at concentrations of 6.3 and 25 mg/mL, these levels were reduced by 50% and 83.2%, respectively (Figure 3A). To demonstrate whether GMP was able to avoid cell damage, we analyzed lipid peroxidation as an index of oxidative damage in cell membranes. The value of LOOH in control HaCaT cells was 4.72 µM, but DNCB stimulus significantly increased LOOH levels to 7.99 µM (Figure 3B). GMP treatment reduced the cell accumulation of DNCB-induced LOOH to 5.79 µM and 4.38 µM, restoring the values to those of control condition with the highest GMP concentration. We also measured the mRNA expression of *HMOX1* to evaluate antioxidant response of the cells. As shown in Figure 3C, DNCB stimulus increased the *HMOX1* gene expression in keratinocytes by 14.6-fold and these levels were reduced by 70.5% and 61.6% when cells were GMP-treated at 6.3 and 25 mg/mL. The three parameters of oxidative stress were similar between control cells and cells treated with GMP in the absence of DNCB, indicating that GMP did not trigger oxidative response in keratinocytes. The antioxidant effect of GMP is related to the decrease in cell death and apoptosis when HaCaT cells are incubated with DNCB in presence of the peptide.

### 3.4. GMP Down-Regulates Gene Expression Associated with Type-2 Inflammatory Response in Keratinocytes

**Figure 4:**
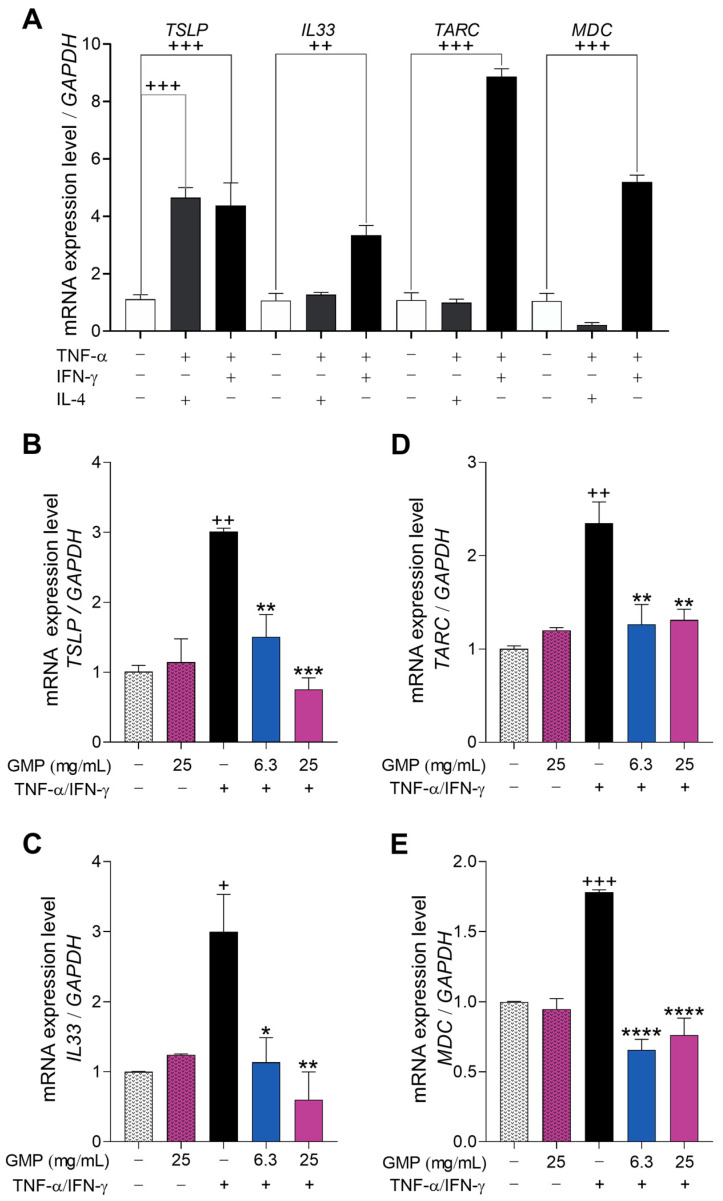
GMP regulates keratinocyte gene expression associated with triggering inflammation in atopic dermatitis (AD). (**A**) In vitro development of an AD model of keratinocytes. HaCaT cells were stimulated with tumor necrosis factor (TNF)-α (10 ng/mL), interferon (IFN)-γ (10 ng/mL) or interleukin (IL)-4 (50 ng/mL) for 24 h. (**B**–**E**) HaCaT cells were incubated with GMP and stimulated with TNF-α/IFN-γ (10 ng/mL). (**B**) *TSLP*, (**C**) *IL33*, (**D**) *TARC* and (**E**) *MDC* gene expression was analyzed by qPCR. n = 3 independent experiments. + *p* < 0.05, ++ *p* < 0.001, +++ *p* < 0.0001 vs. control; * *p* < 0.05, ** *p* < 0.01, *** *p* < 0.001, **** *p* < 0.0001 vs. TNF-α/IFN-γ.

It is known that in response to barrier disruption and exposure to *S. aureus* and allergens, keratinocytes release alarmins and chemokines, such as TSLP, IL-33, TARC, and MDC, that promote the pro-inflammatory type-2 response characteristic of AD [6,9]. To develop an AD model using keratinocytes, we incubated HaCaT cells with the combination of different cytokines that had been previously reported to induce gene expression related to AD [32]. While levels of *TSLP*, *IL33*, *TARC*, and *MDC* mRNAs were significantly increased in HaCaT cells in presence of TNF-α and IFN-γ (Figure 4), only *TSLP* gene expression was up-regulated when cells were stimulated with TNF-α and IL-4. Thus, we chose TNF-α/IFN-γ mixture to stimulate keratinocyte gene expression in the following experiments.

As shown in Figure 4, the increased gene expression of *TSLP* (3-fold; Figure 4B), *IL33* (3-fold; Figure 4C), *TARC* (2.3-fold; Figure 4D), and *MDC* (1.78-fold; Figure 4E) in HaCaT keratinocytes treated with AD-inducing agents was significantly downregulated by GMP treatment to values similar to the control conditions, showing the efficacy of GMP reducing atopic inflammatory responses. In the absence of stimulus, GMP treatment did not modify the expression of type-2 response stimulating cytokines and chemokines in the cells as compared to control conditions.

### 3.5. GMP Modifies Gene Expression Related to Itch and Neurogenic Inflammation

**Figure 5:**
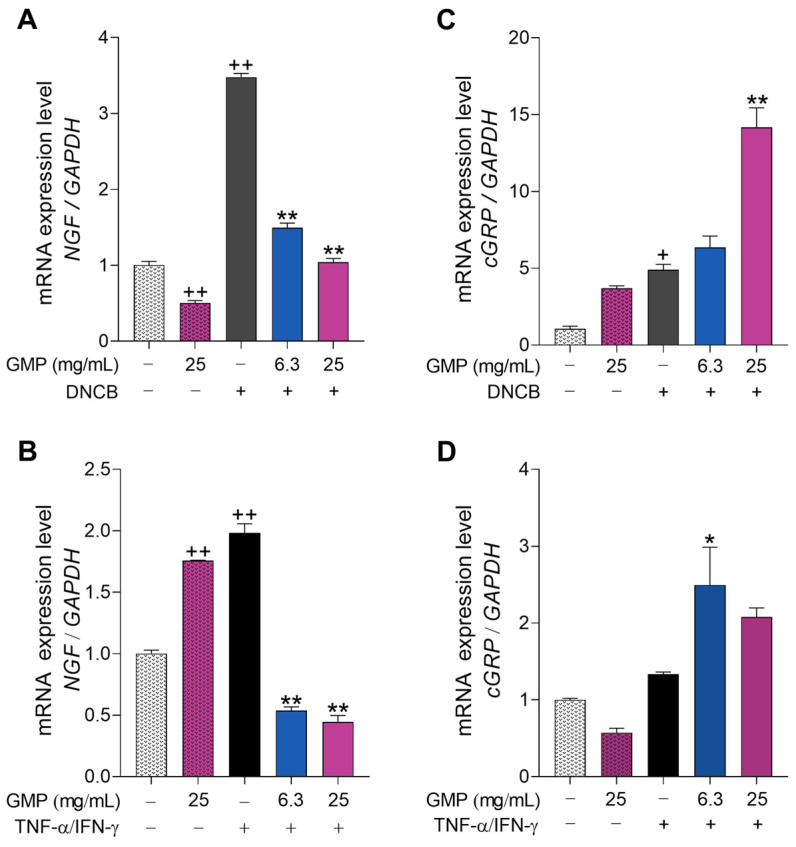
GMP regulates induced NGF and cGRP expression in keratinocytes. HaCaT cells were incubated with GMP and stimulated with: (**A**,**C**) DNCB 15 µM or (**B**,**D**) TNF-α/IFN-γ 10 ng/mL. Gene expression of (**A**,**B**) *NGF* and (**C**,**D**) *cGRP* was analyzed by qPCR. (**A**,**C**) n = 4 independent experiments; (**B**,**D**) n = 3 independent experiments. + *p* < 0.01, ++ *p* < 0.0001 vs. control; * *p* < 0.05, ** *p* < 0.0001 vs. DNCB or TNF-α/IFN-γ.

It has been reported that the symptom most difficult to control in AD therapy is pruritus, which is associated with aggravation of the lesions [33]. Neuropeptides released by keratinocytes, such as NGF and cGRP, play crucial roles in the itching sensation and type-2 inflammation in AD patients [11,33]. Thus, we examined the effect of GMP on *NGF* and *cGRP* gene expression activated by DNCB and cytokines in keratinocytes. *NGF* expression was increased in DNCB- and TNF-α/IFN-γ-stimulated keratinocytes (Figure 5A,B), with DNCB-stimulation inducing a higher effect compared to when cells were incubated with TNF-α/IFN-γ. GMP treatment significantly downregulated mRNA levels of *NGF* induced by both stimuli in HaCaT cells. The expression level of *NGF* was increased in HaCaT cells incubated with GMP in absence of stimulus (Figure 5B), but this upregulation was avoided by GMP in stimulated cells. *cGRP* gene expression was only significantly upregulated when keratinocytes were stimulated with DNCB (4.6-fold; Figure 5C). Nevertheless, when cells were treated with 6.3 and 25 mg/mL GMP before stimulation, induced levels of *cGRP* mRNA were 1.42- and 3.14-fold higher than without GMP treatment (Figure 5C). Although cytokines only slightly upregulated *cGRP* gene expression in HaCaT keratinocytes, the expression level was significantly enhanced with GMP treatment (Figure 5D).

### 3.6. GMP Improves Wound Healing in an In Vitro AD Model of Keratinocytes

**Figure 6:**
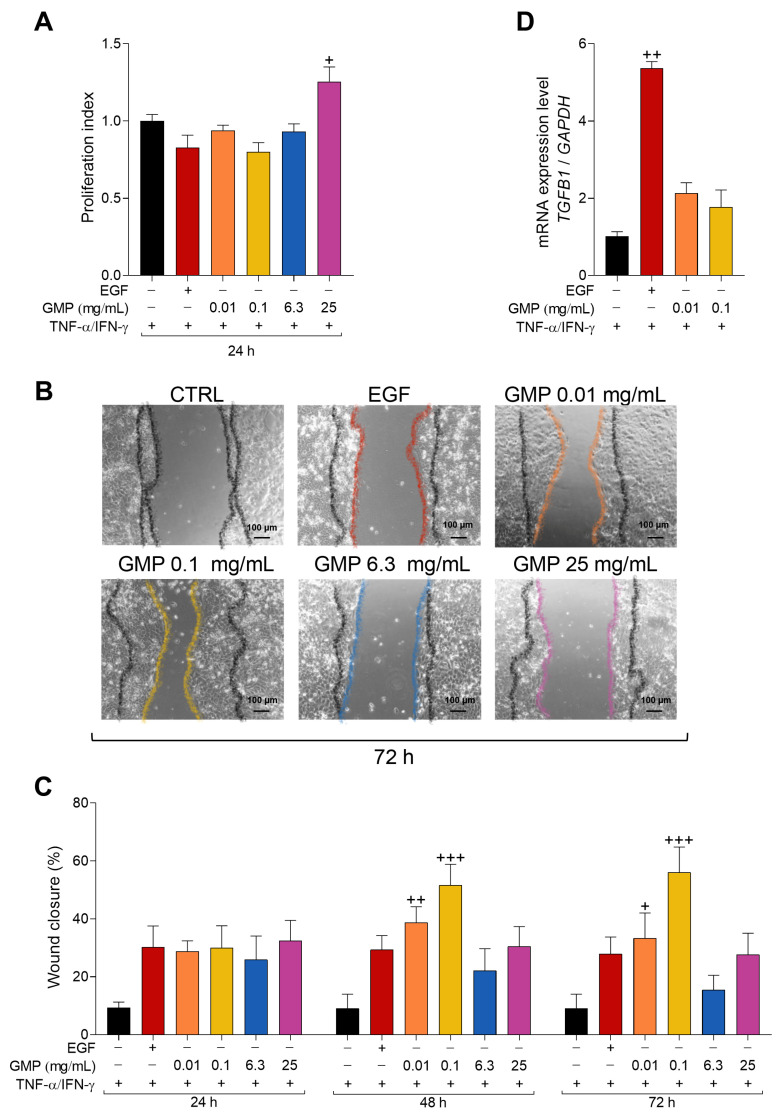
Effect of GMP on proliferation and migration of keratinocytes in an in vitro AD model. (**A**) HaCaT cells were incubated with epidermal growth factor (EGF, positive control) or GMP and stimulated with TNF-α/IFN-γ (10 ng/mL) and the proliferation index was determined. (**B**–**D**) HaCaT cells were cultured until confluent, incubated with mitomycin C, scratched with a pipette tip, re-coated with fibronectin, and incubated with EGF or GMP plus TNF-α/IFN-γ. (**B**) Representative images at 72 h are shown. (**C**) Distance between wound edges was measured and the wound closure percentage at each indicated time was calculated. (**D**) The gene expression of *TGFB1* was analyzed by qPCR at 48 h. (**A**) n = 6, two independent experiments in duplicate; (**C**) n = 12, 4 randomly selected areas per condition in 3 independent experiments; (**D**) n = 3 independent experiments. + *p* < 0.05, ++ *p* < 0.01, +++ *p* < 0.0001 vs. control.

In AD, recurrent wounds are often generated by constant scratching. The re-epithelialization process of wounds requires the proliferation and migration of keratinocytes to cover the naked dermal surface [34], crucial steps that are altered in AD patients [12]. As previously mentioned (Figure 1), HaCaT cell proliferation was significantly increased in the presence of high concentrations of GMP. Thus, we hypothesized that GMP could be beneficial on tissue repair in an AD model of keratinocytes. We first analyzed the effect of low and high GMP concentrations on keratinocyte proliferation under the influence of AD-inducing cytokines. As shown in Figure 6A, GMP at the concentration range from 0.01 to 6.3 mg/mL did not stimulate the proliferation of keratinocytes under the AD microenvironment. GMP at 25 mg/mL slightly but significantly increased the proliferation index of HaCaT cells (*p* < 0.05). Subsequently, we measured the percentage of wound closure at 24 h, 48 h, and 72 h of GMP incubation. Representative images of the wounds at 72 h are shown in Figure 6B. The results showed that at a short incubation time (24 h), GMP at 0.01 to 25 mg/mL increased would closure to a similar extent to EGF, the positive control, reaching a mean percentage of 30% (Figure 6C). When cells were incubated for longer periods of time with GMP, concentrations of 0.01 mg/mL and 0.1 mg/mL significantly increased wound closure as compared to untreated cells (control), reaching percentages of 38.66% and 33.33% at 48 h and 51.66% and 56.08% at 72 h, respectively, while in control conditions the percentage of wound closure was 9.08% at both evaluated times (Figure 6C). In our AD model of keratinocytes, EGF did not significantly stimulate the cell migration at any evaluated time.

During the migration process, the cells undergo an epithelial-mesenchymal transition (EMT), in which TGF-β1 plays an essential role [13]. To evaluate the participation of TGF-β in the stimulated migration of keratinocytes, we analyzed *TGFB1* gene expression in cells incubated with EGF or with GMP concentrations that stimulated cell migration at 48 h after scratching. As shown in Figure 6D, *TGFB1* expression was up-regulated by 4.51-fold in EGF treated cells as compared to control cells. However, *TGFB1* expression was only slightly, but not significantly, increased in cells incubated with GMP.

## 4. Discussion

Keratinocytes have been positioned as crucial cells in the onset, maintenance, and exacerbation of the AD. This chronic and relapsing skin disease negatively impacts the quality of life of patients and their families, and although it mainly occurs in infancy and childhood, it predisposes patients to other allergic diseases later in life. To date, no cure is available for AD. The most widely used treatments are topical corticosteroids and calcineurin inhibitors, even though resolution is temporary, and it is well documented that they present some adverse effects with long-term application [35]. Thus, it is important to investigate new therapies with the potential to modify the disease, as well as to prevent symptoms. Natural bioactive compounds have captured researchers’ attention for this issue. Our group has extensively explored the anti-allergic properties of GMP, showing immunoregulatory, anti-inflammatory, and skin barrier protective activities when orally administered in preclinical models of AD. Nevertheless, there is no information about the effects of GMP on keratinocytes. In the present study, we demonstrate that GMP has no cytotoxic effect on human keratinocytes. Additionally, GMP prevents cell death, apoptosis, and oxidative damage activated by chemical compounds or ROS in human HaCaT cells. In an AD-keratinocyte model, GMP down-regulates the expression of cytokines, chemokines, and neurotrophic factors that trigger the Th2 response, neurogenic inflammation, and pruritus associated to the disease. Under the AD microenvironment, GMP also increases keratinocyte migration, improving wound closures.

GMP represents a good alternative as a protein source in phenylketonuria patients for the elaboration of nutritional supplementation formulas [36]. Different studies suggest the use of GMP as potential therapy in patients that suffer prediabetes or ulcerative colitis [37,38]. Thus, GMP has been extensively explored for general safety issues when orally administered. Using animal models, GMP is reported as non-immunogenic [39]. It is also safe and well-tolerated by humans, with no immunomodulatory effects in healthy adults [40]. Nevertheless, there are no studies about the possible effects of GMP on keratinocytes, either orally or topically administered. In this context, firstly, it should be important to discard GMP toxicity on human keratinocytes. Our results show that GMP does not present cytotoxicity on HaCaT cells when used in a range of concentrations from 0.01 to 25 mg/mL. Previous studies have demonstrated that GMP does not alter the cell viability of human or mouse cell lines at 0.5–2 mg/mL, such as Caco-2/15 human epithelial colonic cells, HepG2 human hepatic cells, and RAW264.7 mouse macrophages [19,41,42]. Our results strengthen the data about the biosafety of GMP.

High level of oxidative stress is involved in the pathophysiology of AD, which eventually induces keratinocyte damage and alters their normal function [43]. The redox imbalance in the cell and the accumulation of ROS have been associated to keratinocyte death [31]. Using activators of oxidative stress in HaCaT cells, we show that GMP has a cytoprotective effect, as it decreases keratinocyte cell death induced by lethal concentrations of both DNCB and H_2_O_2_ in a concentration-dependent manner. Besides, GMP protects keratinocytes against DNCB-induced oxidative damage, decreasing levels of LOOH and NO. Previous results in RAW-264.7 murine macrophages stimulated with H_2_O_2_ or LPS demonstrated that pretreatment with intact or hydrolyzed GMP increases cell viability, reduces apoptosis, decreases oxidative stress levels, and increases the activity of antioxidant enzymes [20,42]. Similar antioxidant effects have been reported to GMP hydrolysates in HepG2 mouse hepatocytes [19]. In both macrophages and hepatocytes, this antioxidant activity was mediated through HMOX-1 expression [19,20]. On the contrary, in our results, GMP down-regulated *HMOX1* expression in keratinocytes activated by DNCB, suggesting that GMP is preventing the cell oxidative response and, thus, the HMOX-1 compensatory expression.

HaCaT cell stimulation with the combination of cytokines TNF-α/IFN-γ or TNF-α/IL-4 induces the differential expression of genes that are up-regulated in the skin of AD patients [32]. As previously reported [32], we show that the stimulation of HaCaT cells with TNF-α/IFN-γ better resemble the gene expression that occurs in keratinocytes during AD. Under the influence of these cytokines, we demonstrated that GMP down-regulates the expression level of *TSLP*, *IL33*, *TARC*, and *MDC* in HaCaT cells. TSLP and IL-33 are two of the predominant activators of ILC2s in AD, which are abundant in skin lesions and, once activated, produce the type 2 cytokines IL-5 and IL-13 [44,45]. TSLP is also involved in dendritic cell activation with the subsequent activation of Th2 cell response [46]. In addition, a direct role of TSLP in Th2 differentiation and activation has been recently described [47]. On the other hand, TARC and MDC are chemokines elevated in serum and associated with Th2 lymphocyte attraction and severity of lesional skin in AD patients [6]. We previously reported that when GMP was administered orally in rats, the expression level of Th2 cytokines IL-4, IL-5, and IL-13 was decreased in AD-lesions, which was related to the improvement in clinical signs [21]. Thus, the down-regulatory effect of GMP on the pathological and dominant Th2 immune response might be mediated, at least in part, through decreasing the expression of TSLP, IL-33, TARC, and MDC in keratinocytes. It is also important to consider that inflammatory cytokine expression in keratinocytes, such as IL-33, can be downregulated by metabolites of skin microbiota, and that AD pathogenesis is associated with skin microbial dysbiosis, characterized by a marked reduction in microbial diversity with increment of Staphylococci abundance [1,48]. As orally administered GMP prevents *S. aureus* colonization in a rat model of AD and GMP has been extensively reported as prebiotic [18,22], future works might explore if GMP impairs growth or adhesion of *S. aureus* in keratinocytes in vitro.

Keratinocytes are key source of NGF, a neurotrophic factor that participates in neurogenic inflammation and pruritus [49]. NGF is also involved in the excessive sprouting of cutaneous sensory nerve fibers characteristic of AD [50]. Our results show that GMP reduces the increased expression of NGF induced by cytokines or DNCB in HaCaT cells. These results are in accordance with the anti-pruritic and anti-inflammatory effect of GMP in pre-clinal models of AD [21]. Nevertheless, pruritus can also be triggered by cytokines. An elevated expression of IL-33 is reported in AD lesions of adult patients, which is significantly associated with the itch [51]. Additionally, TSLP released by keratinocytes acts directly on a subset of sensory neurons to trigger robust itch behaviors in animal models [52]. Thus, the decreased expression of *IL33* and *TSLP* in our AD model of keratinocytes might be also causing the abolishment of pruritus in rats prophylactically treated with GMP that has been previously reported [21]. We propose that GMP might be regulating the expression of *NGF*, *TSLP,* and *IL33* through its antioxidant activity. This suggestion is supported by studies showing that ROS production in keratinocytes is linked to the upregulation of mRNA levels of the aforementioned biomarkers of inflammation in AD [10]. On the other hand, we show that HaCaT cells only up-regulate *cGRP* expression when cells were stimulated with DNCB, but not with cytokines. However, in both conditions, GMP significantly enhanced *cGRP* expression. The role of cGRP in AD pathophysiology is controversial. Classically, it is considered to be a neuropeptide involved in skin neurogenic inflammation, participating in pruritus and mainly in vasodilation [11]. Most studies that analyze cGRP in skin are focused on evaluating the density of cGRP-positive fibers in AD patients, observing an increase or no change in cGRP innervation in lesional skin [53,54]. When skin homogenates of AD mice are evaluated, significantly lower cGRP concentration is found as compared to control mice [55]. In accordance, lower cGRP plasma level is found in patients with AD, which is normalized after treatment [56]. These results might suggest a possible immunomodulatory role of cGRP during AD. In this context, the consequences of increased expression of *cGRP* mRNA in keratinocytes induced by GMP must be exhaustively analyzed in future studies. It is also possible that GMP exerts beneficial effects in experimental AD despite increasing the expression level of cGRP in keratinocytes, because GMP inhibits activation of mast cells [23], cells that, in response to cGRP, release mediators that trigger pruritus and neurogenic inflammation.

In a mouse model that resembles human AD, animals present a delayed wound closure process, and although there is an increased proliferation of keratinocytes, the cells do not migrate efficiently, resulting in a delayed re-epithelization [12]. Based on these observations, we decided to evaluate the effect of GMP on the proliferation and migration of AD keratinocytes in our experimental model. Our results showed that GMP at high concentrations increased keratinocyte proliferation without modifying the wound healing process, and at low concentrations did not affect cell proliferation but increased keratinocyte migration. It is important to highlight that the proliferative effect of GMP on HaCaT cells reported under the control condition (Figure 1) was almost completely lost when cells were under an AD microenvironment (Figure 6A). In the present work, we report for the first time a benefic potential effect of GMP on wound closure. Other bioactive natural substances with anti-inflammatory and antioxidant properties, such as quercetin, also present a potential therapeutic application in wound healing in AD [57]. EMT is a process that occurs in cells as they acquire migratory behavior and is triggered by TGF-β signaling [58]. Particularly, TGF-β promotes migration of HaCaT cells, which is potentiated by EGF [59]. Under our AD microenvironment, EGF, but not GMP, increased TGF-β mRNA expression in HaCaT cells, suggesting that enhancement of migratory activity induced by GMP is not TGF-β-mediated. The anti-allergic effect of oral treatment with GMP is related to systemic TGF-β production, but also to down-regulation of TGF-β expression in the asthmatic lung tissue [23,60]. In addition, the anti-inflammatory and immunoregulatory effect of orally administered GMP on experimental colitis models has been associated with down-regulation of TGF-β expression in colonic tissue, but also with an increase in TGF-β-mediated signaling [61,62]. Thus, although GMP can modify TGF-β expression in other cells, it apparently does not alter its expression in migratory AD-keratinocytes.

**Figure 7:**
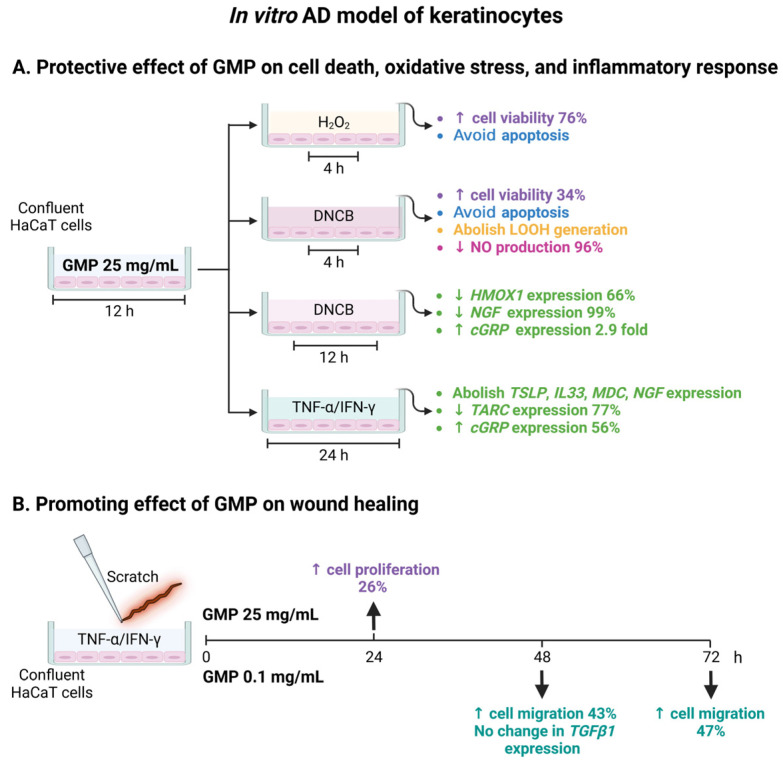
Schematic representation of GMP effects on viability, inflammatory and oxidative response, and the re-epithelization capacity of keratinocytes in an in vitro AD model. Created with BioRender.com (accessed on 3 May 2023).

In summary, we show that GMP at high concentrations presents cytoprotective, anti-inflammatory, and antioxidant activities, and promotes keratinocyte proliferation under an AD microenvironment. Additionally, GMP at low concentrations induces cell migration (Figure 7). Although GMP might be incorporated to topical formulations at different concentrations to enhance a particular bioactivity, GMP at high concentrations could present wound healing effects in vivo in AD lesions, as cell migration is only one step in the complex process of re-epithelialization [34], and excessive ROS levels are proposed as detrimental in the chronic and non-healing wounds in vivo [63]. Thus, in vivo assays are required to define the optimal GMP concentration to be topically applied in AD patients.

## 5. Conclusions

In conclusion, these results suggest that GMP protects from death, inflammation, and oxidative stress, and stimulates wound healing in an AD model of keratinocytes. This work reinforces the evidence that GMP may be a potential therapeutic candidate for AD, highlighting its beneficial effects on keratinocytes. The limitation of this work is that it comprises an in vitro study in which keratinocytes are alone and under culture conditions, without the contact with other skin cells, molecules, and microbiota that occur during AD and could modify keratinocyte action. Our results support further studies to confirm the bioactivity of GMP in keratinocytes in vivo under the influence of other skin components, as well as to stablish the optimal GMP concentration for topical application, using equivalent skin systems, organoids, biopsies of human skin, or animal models.

## Figures and Tables

**Table 1:** The primers used in this study.

Target Genes	NCBI Access Number	Primers
*TSLP*	NM_033035.5	Fw: ATGTTCGCCATGAAAACTAAGGC
		Rv: GCGACGCCACAATCCTTGTA
*IL33*	NM_033439.4	Fw: GGAGTGCTTTGCCTTTGGTA
		Rv: CATTTGAGGGGTGTTGAGAC
*CCL22/MDC*	NM_002990.5	Fw: GCACTCCTGGTTGTCCTCGT
		Rv: GACGTAATCACGGCAGCAGA
*CCL17/TARC*	NM_002987.3	Fw: GTACTTCAAGGGAGCCATTC
		Rv: CACTCTCTTGTTGTTGGGGT
*HMOX1*	NM_002133.3	Fw: AAGACTGCGTTCCTGCTCAAC
		Rv: AAAGCCCTACAGCAACTGTCG
*cGRP/CALCA*	NM_001033952.3	Fw: TCTAAGCGGTGCGGTAATCTG
		Rv: CAGTTTGGGGGAACGTGTGA
*NGF*	NM_002506.3	Fw: TGTGGGTTGGGGATAAGACCA
		Rv: GCTGTCAACGGGATTTGGGT
*TGFB1*	NM_000660.7	Fw: CTCCCCACCACACCAGCCCT
		Rv: GCCACAGCAGCGGTAGCAGC
*GADPH*	NM_002046.7	Fw: ATCCCATCACCATCTTCCAG
		Rv: GGCAGAGATGATGACCCTTT

Fw, forward; Rv, reverse.

## Data Availability

The data presented in this study are available on request from the corresponding author.
